# Postnatal imaging of conjoined twins: a customized multimodality approach

**DOI:** 10.1007/s00247-023-05709-3

**Published:** 2023-07-19

**Authors:** Shaimaa Abdelsattar Mohammad, Amr Abdelhamid AbouZeid, Khaled A. Ahmed, Abeer Maghawry Abd-Elhamed, Leila M. Rawash Eldieb

**Affiliations:** 1 Department of Diagnostic and Interventional Radiology and Molecular Imaging, Faculty of Medicine, Ain Shams University, Cairo, Egypt; 2 Department of Pediatric Surgery, Faculty of Medicine, Ain Shams University, Cairo, Egypt

**Keywords:** Conjoined twins, Diagnostic imaging, Magnetic resonance imaging, Three-dimensional imaging, Three-dimensional printing, Virtual reality

## Abstract

Medical imaging plays a crucial role in the management of conjoined twins. The role of imaging is to explore the anatomy, outline the shared organs and determine whether surgery is feasible. It also serves as a roadmap for successful separation. Additionally, imaging helps with counseling parents about prognosis. This review aims to illustrate recent advances in different imaging modalities and their role in the management of the various types of conjoined twins with an emphasis on relevant tips for optimal imaging.

**Graphical abstract:**

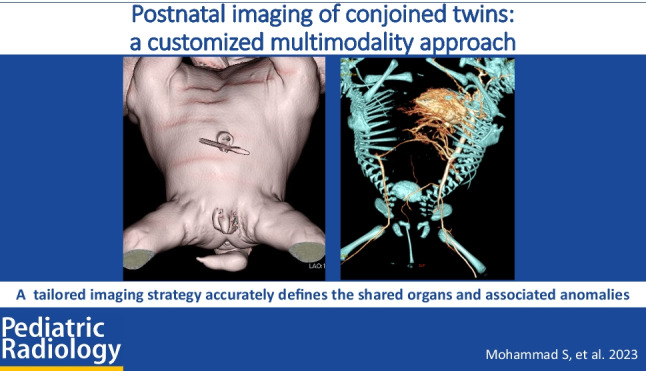

**Supplementary Information:**

The online version contains supplementary material available at 10.1007/s00247-023-05709-3.

## Introduction

Conjoined twinning may be one of the most fascinating conditions that a doctor will face in their medical career. Its incidence varies from 1 in 50,000 to 1 in 200,000 births with reported female predominance (3:1) [1]. Conjoined twinning is considered a unique complication of monoamniotic gestation. It is postulated that conjoined twins result from the failure of complete splitting of a single fertilized egg into two separate embryos (fission theory). However, another theory proposes that conjoined twining may result from secondary fusion of two separate mono-ovular embryos (fusion theory) [2–4]. Although the majority of conjoined twins either die at birth or are stillborn, some do survive to be separated [5, 6].

In the antenatal period, the diagnosis of conjoined twins depends on ultrasound (US) and magnetic resonance imaging (MRI) because both methods avoid ionizing radiation. This is important for prenatal counseling, planning and proper obstetric management. Antenatal diagnosis is usually by US as early as 12 weeks of gestation. However, a thorough evaluation of visceral fusion only becomes possible at 20 weeks of gestation. Conjoined twinning is suspected when there is a single placenta with a single umbilical cord having more than three vessels; a fixed relative position of both fetuses without intervening amniotic membrane is another clue to the diagnosis. The fetal bodies may be clearly inseparable at obvious fusion sites and the limbs may be fewer than expected [5]. Because of the increased through transmission of US waves in the amniotic fluid, echocardiography is better performed in utero. After delivery, the air-filled lung may hinder the proper evaluation of the cardiac chambers, especially in twins with thoracic fusion [1].

The imaging algorithm differs postnatally according to the site of fusion. The role of imaging is to explore the anatomy, outline the shared organs and determine whether surgery is feasible. It also serves as a roadmap for successful separation. Additionally, imaging helps with counseling parents concerning prognosis [7]. This article aims to illustrate the evolution and recent advances of different imaging modalities and postprocessing techniques and their role in the management of the various types of conjoined twins with an emphasis on relevant tips for optimal imaging. This review may be used as a guide for clinical radiologists, especially when facing such rare and complex cases for the first time.

## Classification of conjoined twins

The first clay sculptures with varying degrees of facial and cranial duplications were produced 3,000 years ago. They were discovered during the excavation of the Mexican village of Tlatilco [8]. However, the first well-documented case of conjoined twins was not until 1811 (the Siamese twins) [9]. Since then, there have been a number of attempts to classify conjoined twins.

**Fig. 1:**
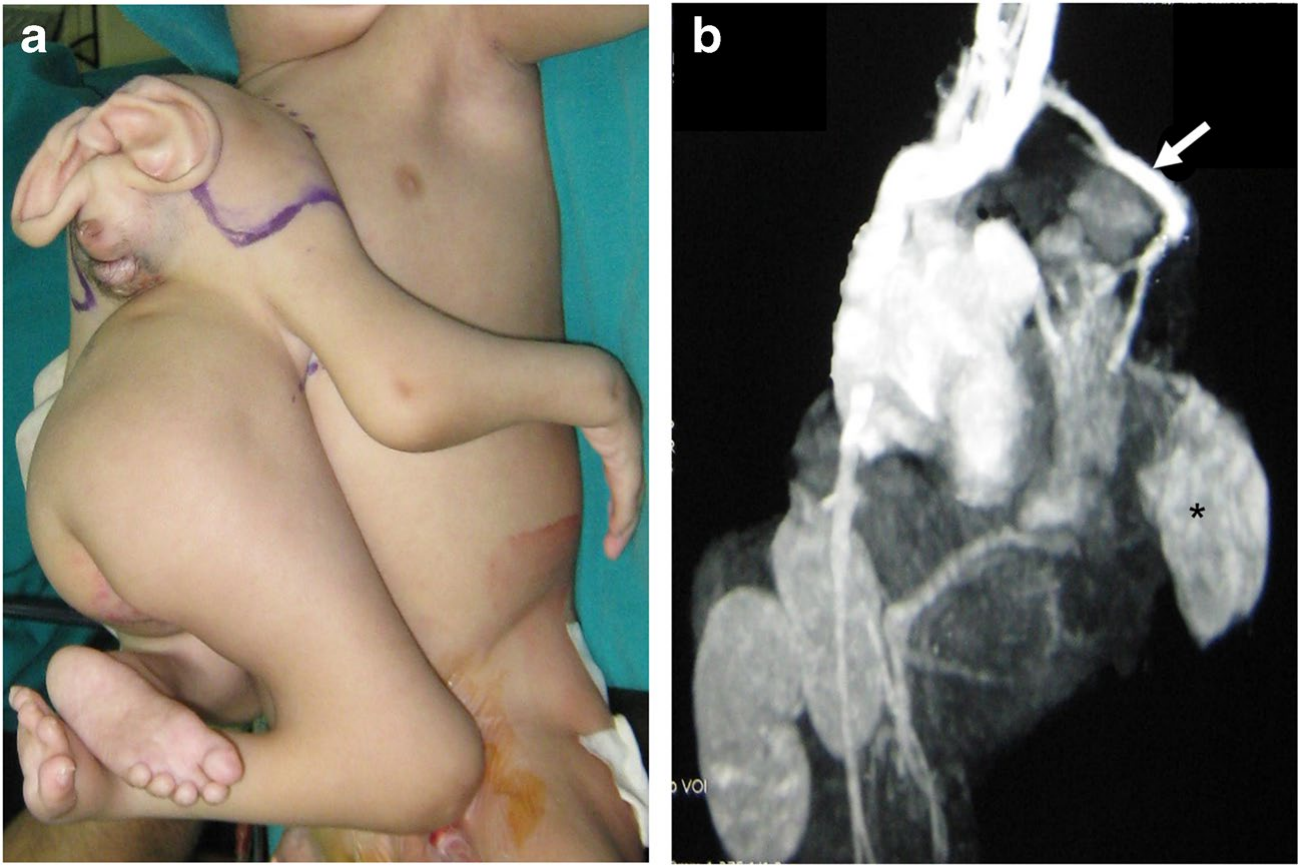
15-month-old heteropagus conjoined twin girls. **a** A photographic images shows the parasitic twin attached to the anterior chest wall of the autositic twin. The parasitic twin has a deformed cranium and incomplete trunk with four limbs. **b** Maximum intensity projection computed tomography angiography shows an anomalous vessel (*arrow*) supplying the parasitic twin which originates from the subclavian artery of the autosite. Note the kidney of the parasitic twin (*asterisk*)

**Fig. 2:**
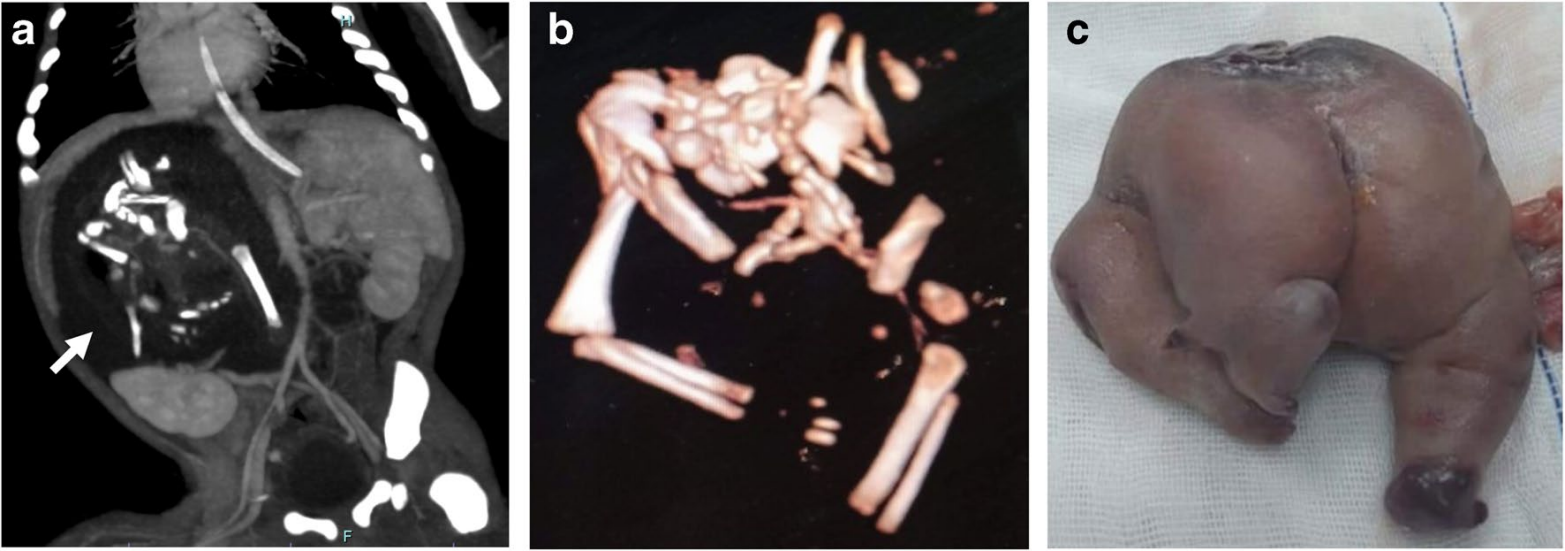
A 1-month-old boy presented with abdominal swelling which proved to be a parasitic twin (fetus in fetu). **a** Coronal reformatted computed tomoagraphy image of the abdomen and pelvis (soft tissue window) of the autosite shows the bony skeleton of the parasitic twin within a fatty mass (*arrow)*. **b** 3-dimensional reformat of the skeleton nicely demonstrates the presence of a dysplastic sacrum and bones of the pelvis and lower limb at variable degrees of development. **c** Post-operative photographic image after excision of the parasitic twin

Conjoined twins may be classified according to the pattern of fusion, whether symmetric or asymmetric. Conjoined twins are called asymmetric when one is completely formed (autosite), while the other has missing body portions (parasitic twin). The parasitic twin may remain attached to the exterior of the other, complete twin, resulting in heteropagus twinning (Fig. 1), or it may be within the body of the autosite fetus when it is termed “fetus in fetu” (Fig. 2). Some authors consider the “fetus in fetu” as a well-organized teratoma. However, there are many concerns about this assumption. Teratoma commonly occurs in the sacrococcygeal region, while the “fetus in fetu” predominantly occurs in the upper retroperitoneum. Unlike presacral teratoma, malignant transformation is extremely rare in the “fetus in fetu.” It has been documented that the identification of the vertebral column is a good discriminator for the identification of “fetus in fetu” [10]. Acardius acephalus is a special entity of asymmetric twins devoid of a heart and head. The circulation and nutrition are secured by marginal placental vessels that connect to the autosite fetus [11].

Symmetric conjoined twins have the same sex and general features. Spencer stratified symmetric conjoined twins into eight main types according to the type of union (ventral, dorsal or lateral) and according to the predominantly fused sites [12]: craniopagus, thoracopagus, omphalopagus, ischiopagus, etc. The suffix “pagus” means fixed. Table 1 summarizes the different types of symmetric twins, their subtypes and commonly shared organs. This unified system of terminology was developed to make it easier to predict the site of fusion in these patients [12]. However, it may not always be possible to categorize them precisely, because there is often some degree of overlap between different types (e.g., thoraco-omphalopagus; omphalo-ischiopagus) [13]. From a clinical standpoint, identifying the actual shared organs and related anomalies is more crucial than defining the precise classification. Identifying the shared organs determines the pertinent expertize required within the multidisciplinary team, the feasibility of surgery and improves surgical outcome.

**Table 1:** Types of conjoined twins with symmetric fusion

Type of fusion	Major clinical types/incidence	Definition/maximum site of fusion	Subtypes	Remarks
Ventral fusion	Cephalopagus 11%	Twins are fused from the head down to the umbilicus. These twins are usually nonviable	1. Symmetric (two well-formed faces on opposite sides of the head) 2. Asymmetric (one well-formed face and one hypotrophic face on opposite sides) 3. Deradelphous (one midline face) 4. Deradelphous diprosopous (one midline face with duplication of some facial features)	Usually, they have one brain with possible duplication of other structures such as the cerebellum Usually, they share the upper gastrointestinal tracts down to the site of Meckel’s diverticulum, while the large bowels and rectums are separate
	Thoracopagus 47–71%	Twins are essentially fused at the thorax. Usually, fusion extends down to the umbilicus (thoraco-omphalopagus)	1. Separate hearts and pericardium 2. Separate hearts but common pericardium 3. Fused atria and separate ventricles 4. Fused atria and ventricles	The fusion involves the sternum, diaphragm, upper abdominal wall and liver Sometimes, there is upper gastrointestinal tract fusion down to the site of Meckel’s diverticulum Major cardiac anomalies are nearly always present
	Omphalopagus 20%	Twins are essentially fused at the abdomen. Hepatic fusion is invariable with no cardiac sharing		There may be variable degrees of small bowel sharing Twins with a single duodenum have a higher incidence of a shared biliary system
	Ischiopagus 6–11%	This term describes twins who are essentially fused at the pelvis, usually also with lower abdominal fusion	Commonly, the fusion is ventral (face to face); less commonly, the fusion may be “end to end” with 180° inclination angle between the body axes of both twins There may be hypodevelopment of the lower extremities with missing lower limbs Ischiopagus twins may be further subclassified according to the number of lower limbs as tetrapus, tripus and bipus with four, three or two lower limbs, respectively)	Each twin has anterior pubic diastasis (open pelvic ring like in exstrophy) Facing each other, each pelvis constitutes a hemicircle that are joined together by two cartilaginous joints between opposite pubic bones forming a common pelvic cavity The external genitalia and anus are always shared They may have shared or separate urinary bladders Fusion may extend up to the diaphragm with variable hepatic and gastrointestinal fusion
Dorsal fusion	Craniopagus 2%	Twins are fused at any segment of the meninges, bony cranium and skull except foramen magnum. They have separate faces and brain Twins do not have any shared thoracic, abdominal or pelvic organs	1. Total craniopagus (there are large cranial unions with two brains) 2. Partial craniopagus (there are smaller unions limited to the dural or there is leptomeningeal fusion) Both are further subclassified into angular or vertical	Cortical fusion and shared cerebral venous sinuses may be seen in up to one third of cases Majority have associated cardiovascular, gastrointestinal, genitourinary, craniofacial, and neurologic abnormalities
	Pygopagus 18–28%	Twins are dorsally fused mostly at the sacrococcygeal and perineal regions		Twins usually share a common anus with or without a common rectum The genitourinary tract is less frequently involved The spinal cords are usually separate with or without communication of both thecal sacs The degree of spinal cord fusion determines the possibility of separation
	Rachipagus Less than 1% (rarest type)	Twins are fused dorsally above the sacrum		The union may involve the occiput and vertebral column in variable degrees One twin may be parasitic
Lateral fusion: (side-by-side fusion)	Parapagus 28%	Twins have shared umbilicus, abdomen, and pelvis. The shared pelvis usually has a single symphysis pubis and one or two sacra	1. Dithoracic parapagus: the union is limited to the abdomen and pelvis 2. Dicephalic parapagus: the union involves the entire trunk. They have single heart with cardiac anomalies 3. Diprosopic parapagus: one trunk and one head with two faces. two faces are on the same side of the head	They usually have associated genitourinary and anorectal anomalies Usually, there is underdevelopment of the upper and lower extremities with missing limbs. Parapagus twins may have two, three, or four arms, and only two or three legs

## Determination of spatial relationship and selection of postnatal imaging studies

The determination of the spatial relationship of conjoined twins is a major aspect of precise imaging interpretation. During imaging, ventrally fused twins are placed on their sides. We must identify which side should be posterior in the resting “standard” position; there is usually some tilting of their trunks such that the abdomen and umbilicus are facing upwards. In this resting position, we may assign the radiological right twin as twin A and the other as twin B. If lateralizing characteristics are not identified (e.g., in some craniopagus twins), an olive oil capsule can be placed on the cheek of one twin during scanning as a reminder. Twins should have the same orientation during all subsequent imaging investigations [14].

The selection of imaging studies depends on the required organ system to be investigated and the anatomical site of fusion. However, postnatal echocardiography and cranial and abdominal US are required for screening of all conjoined twins irrespective of their type. Abdominal US can demonstrate anomalies in the urinary system and detect hydronephrosis or renal fusion [15]. Chest and abdominopelvic radiography is also required for an overall evaluation. Chest radiographic images can demonstrate lobar consolidation, collapse, hypoplasia or associated diaphragmatic hernia. Abdominal radiography may depict bowel obstruction requiring interim management [4, 5].

**Fig. 3:**
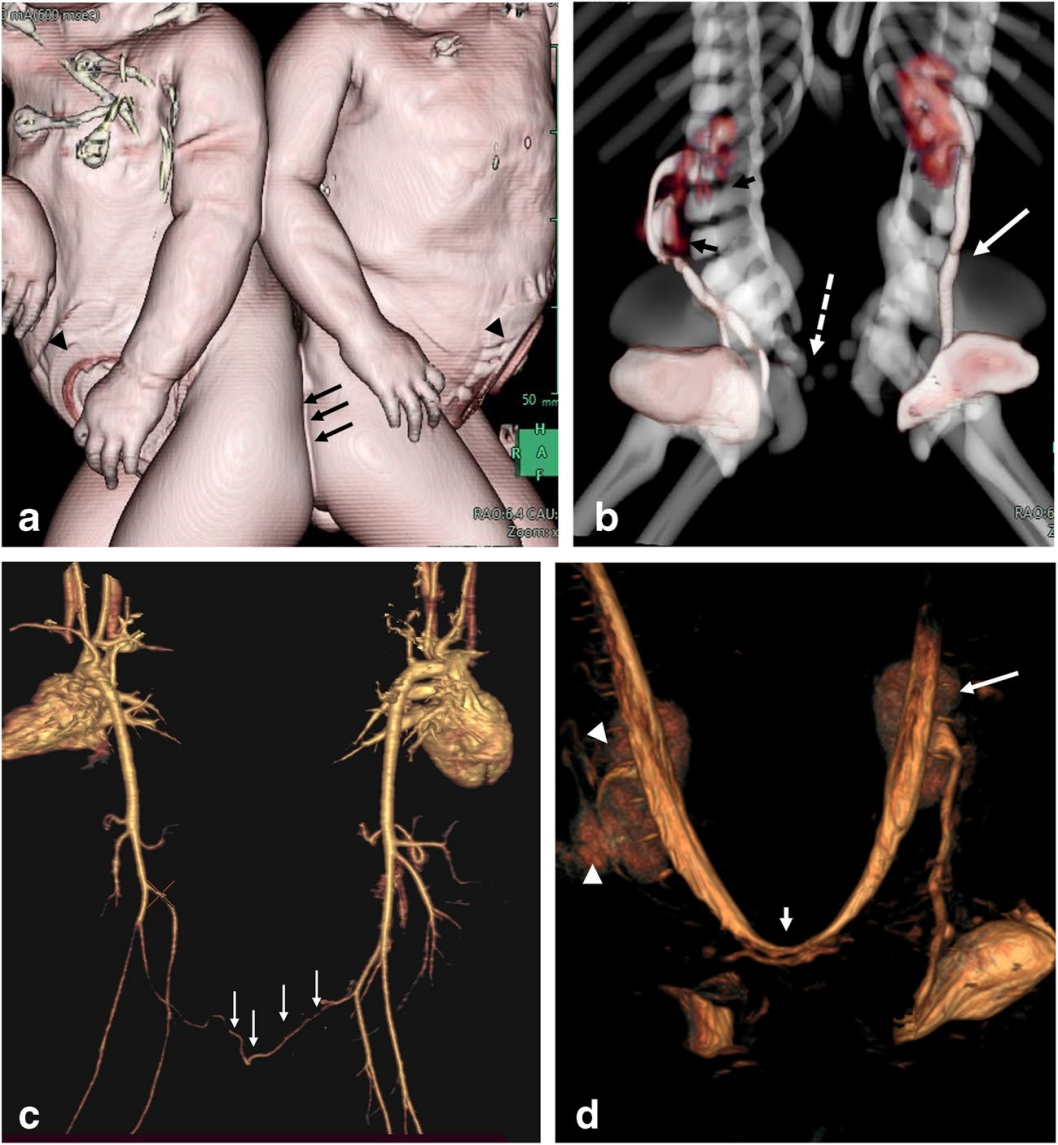
16-year-old pygopagus conjoined twin girls. **a** 3-dimensional (D) surface-rendered image reveals the site of fusion (*arrows*). Note the colostomy sites in both twins (*arrowheads*). **b** 3-D multimask image extracted from a computed tomography (CT) study in the excretory phase shows limited sacral fusion (*broken arrow*). The right twin has crossed fused kidneys (*black arrows*), while the left twin has a single kidney with crossed ectopia (*white arrow*). **c** CT angiography in the arterial phase (both twins injected simultaneously) shows arterial communication (*arrows*). **d** Maximum intensity projection of the coronal fat suppressed T2-weighted magnetic resonance image reveals that both thecal sacs are continuous through a narrow channel (*short arrow*). Note the single kidney of the left twin (*arrow*) and crossed fused ectopia of the right twin (*arrowheads*)

Following initial screening with US and conventional radiography, computed tomography (CT) and MRI are the investigations of choice to show the anatomical region of concern according to the site of conjunction (Fig. 3). Dynamic post-contrast CT is performed after contrast injection in each twin on consecutive days. However, conjoined twins with limited pelvic fusion (pygopagus twins) may undergo CT with simultaneous contrast injection (Fig. 3). Once they have been acquired, the images are transmitted into the workstation for additional post-processing and multiplanar reconstruction. Arterial and venous phases are crucial for the demonstration of vascular anatomy and possible circulatory sharing. Multiple delayed phases are important for demonstrating urologic anomalies.

**Fig. 4:**
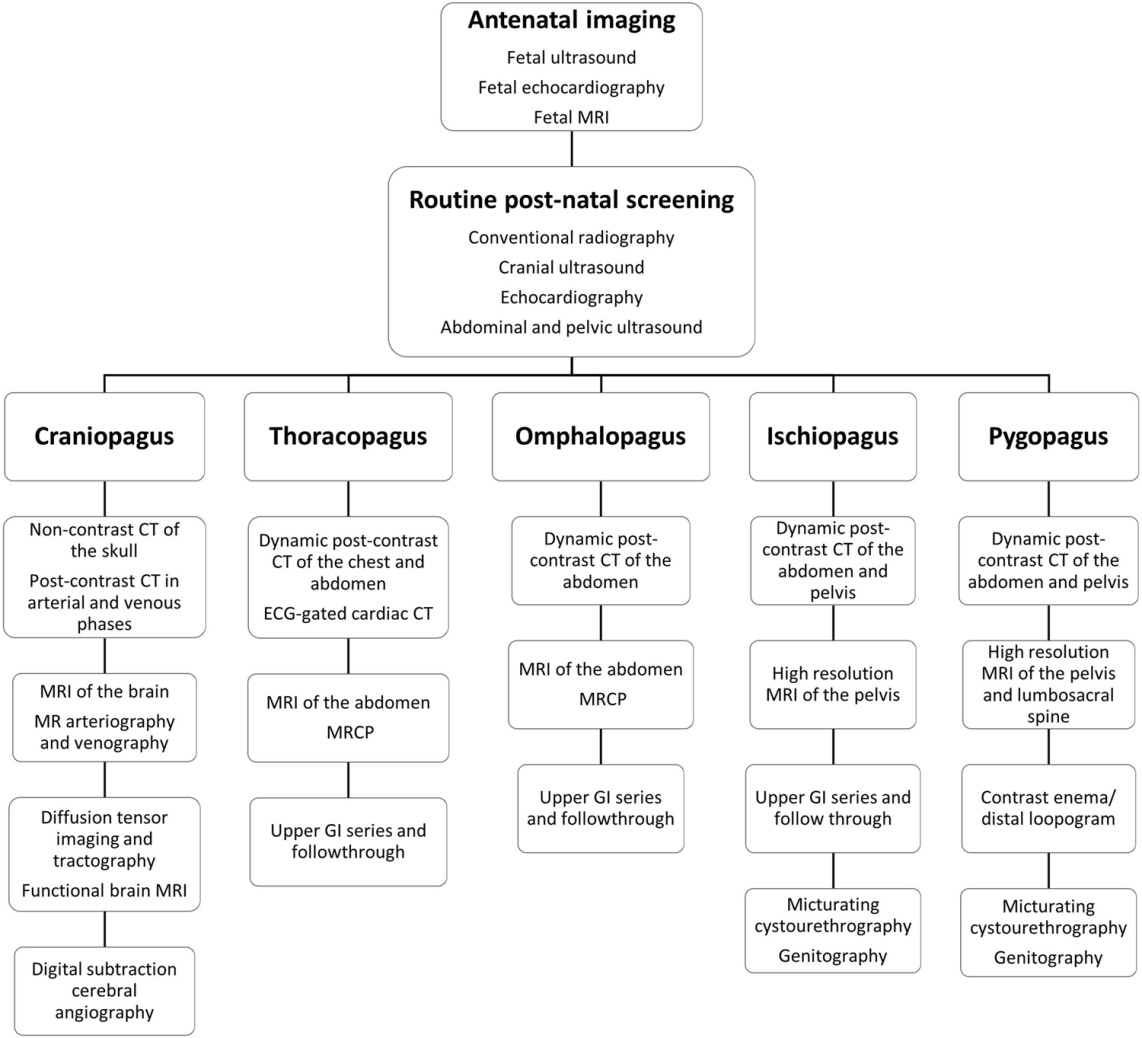
Flowchart depicting the sequence of imaging modalities in relatively common types of conjoined twins. *CT* computed tomography, *ECG* electrocardiography, *GI* gastrointestinal, *MRCP* magnetic resonance cholangiopancreatography, *MRI* magnetic resonance imaging

Compared to CT, MRI has a higher soft tissue resolution. It is recommended for better evaluation of the brain, spine and pelvic anatomy. However, the examination should be performed under general anesthesia. Two anesthetic teams, two anesthetic machines and MRI-compatible monitors are required. To improve the signal-to-noise ratio, a phased-array surface coil spanning both twins should be employed. Imaging algorithms for relatively common types of conjoined twins are provided in Fig. 4.

When one twin is about to pass away, emergency separation may be the only option in order to save the other. In such circumstances, there is no time for detailed preoperative imaging and conventional radiography and US may suffice.

The organ system-based selection of imaging modalities in different types of conjoined twins will be discussed in the following sections and is summarized in Table 2.

**Table 2:** Organ system-based guidelines for imaging of conjoined twins

General imaging guidelines	• Twins should have the same orientation during all imaging investigations • CT is performed by using multislice thin section machines • The dose of contrast agent is calculated according to the combined weight by using pump injector (contrast dose and iodine concentration may vary by institution) • Each twin should be injected on a separate day. Twins with limited pelvic fusion (as pygopagus twins) may undergo CT with simultaneous contrast injection • The images are transmitted into the workstation for additional post-processing and multiplanar reconstruction • MRI usually needs general anesthesia (General anesthesia is better than deep sedation) • Two anesthesia teams should be available throughout the study • A phased array surface coil is used to cover both twins during MRI examination
Twins with fusion of the cranium and vertebral column	Imaging aims for evaluation of the central nervous system • Non-contrast CT with 3-D reconstruction of the bony calvarium and vertebral column • CT cerebral arteriography and venography • MRI standard sequences in addition to high resolution, 3-D heavily T2-WI volumetric sequences of the brain and spinal cord • Non-contrast MR arteriography and venography • Dynamic post-contrast MR angiography of the cerebral circulation • Functional MRI and Diffusion tensor imaging
Twins with thoracic fusion`	Imaging aims for evaluation of the cardiac and pulmonary systems • Dynamic post-contrast study in the arterial and venous phases • ECG-gated post-contrast CT for cardiac anomalies • 3-D reconstruction of the thoracic cage
Twins with abdominal fusion	Imaging aims for evaluation of the hepatobiliary and gastrointestinal systems and kidneys • Post-contrast study in arterial and venous phases for demonstration of shared arteries, or veins and demonstration of the surgical plane of hepatic separation • MRCP and HIDA scan: to rule out biliary fusion if suspected • Upper gastrointestinal series and follow through
Twins with fusion of the pelvis and perineum	Imaging aims for evaluation of the urogenital systems, distal gastrointestinal system and perineum • Post contrast study in arterial and venous phases with acquisition of multiple delayed phases to demonstrate urologic anomalies especially of the urinary bladder and ureters • 3-D reconstruction of the bony pelvis • High resolution T2-weighted image in axial and coronal planes with and without fat suppression for demonstration of the associated genital, urologic anomalies, and pelvic floor anatomy • Distal loopogram (if colostomy is present) for demonstration of anorectal anomalies (e.g., rectourethral fistula) • Micturating cystourethrography demonstrate of reflux and other associated renal anomalies especially if there is urinary tract dilatation (detected by ultrasound)

*CT *computed tomography; *ECG *electrocardiogram, *HIDA *hepatobiliary iminodiacetic acid, *MRCP *magnetic resonance cholangiopancreatography, *MRI* magnetic resonance imaging, *3-D, *three-dimensional

## Central nervous system

**Fig. 5:**
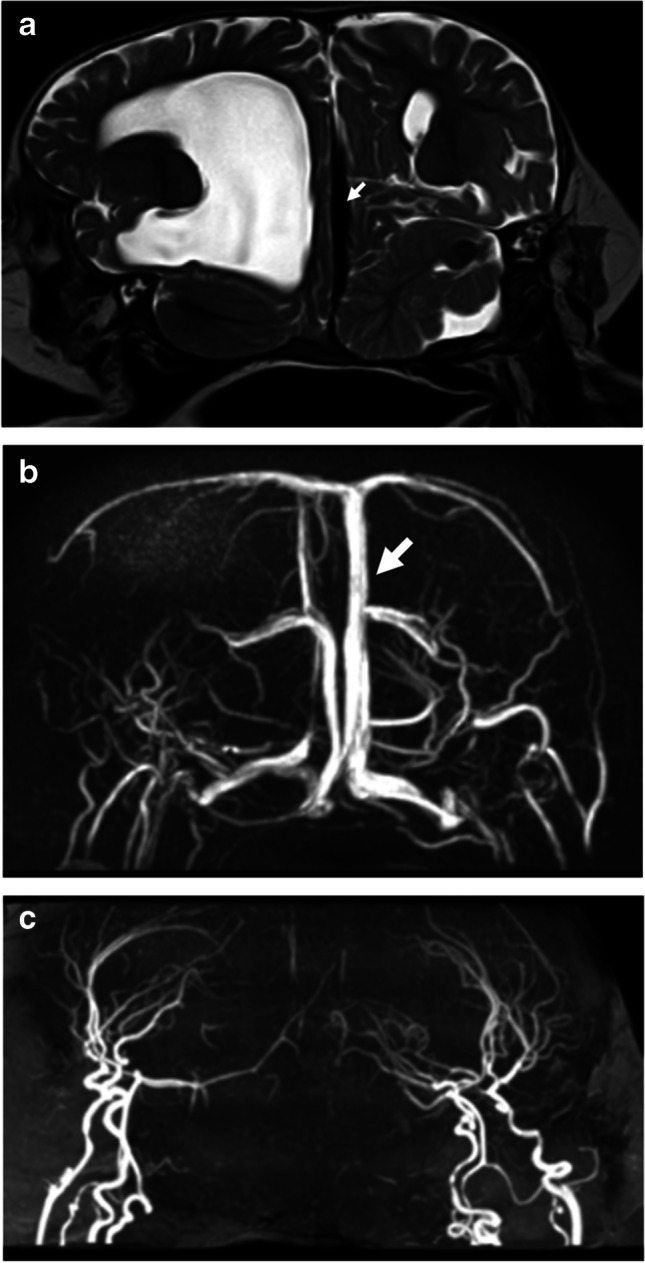
Magnetic resonance images in 2-year-old craniopagus conjoined twin girls. **a** Coronal T2-weighted image shows cranial fusion with two separate brains with an intervening common venous sinus (*arrow*). **b** Coronal magnetic resonance venography image confirms sharing of the superior sagittal sinus (*arrow*). **c** Coronal magnetic resonance arteriography image reveals no significant arterial sharing

MRI is superior to CT in the demonstration of neural fusion, allowing detailed assessment of the brain anatomy, myelination and associated anomalies. The presence of small parenchymal bridges should be evaluated especially at locations without a visible dural shelf. The presence of eloquent areas in these bridges should be emphasized [14]. Eloquent areas represent areas of the brain that have a well-defined function the damage of which will cause devastating neurological deficit, e.g., primary motor cortex, primary somatosensory cortex, primary visual cortex, primary auditory cortex and Broca’s and Wernicke’s areas [16]. In older children, hemispheric language dominance can be assessed before surgery using functional MRI. Diffusion tensor tractography can demonstrate the continuity of white matter tracts [14, 17]. However, the distorted anatomy may render the interpretation of tractography challenging due to the altered color hue of the fiber tracts. MRI can demonstrate the extent of thecal sac or spinal cord sharing (Fig. 5). The site of spinal cord fusion is described as V-, U- or Y-shaped. In U-shaped fusion, it is more challenging to determine the twins’ cleavage planes. In this instance, a triggered electromyogram is used intraoperatively for functional midline cleavage [18].

Non-contrast CT of the skull with 3-dimensional (D) reconstruction is required for the demonstration of the extent of bony cranial and vertebral fusion (supplementary material 1). Subsequently, CT angiography performed in the arterial and venous phases is important to exclude the possibility of shared arteries and veins, respectively. CT angiography is more superior than MRI angiography in this instance [16]. CT angiography can provide a perfusion image that identifies areas of the brain of one twin that are supplied by the other, with clear demonstration of leptomeningeal vascularity. Although the cerebral arterial supply is usually separate in craniopagus twins, the major dural sinuses are usually shared (Fig. 5). There may be cross circulation from the arteries and veins. These findings are crucial to neurosurgeons when planning the surgical procedure [14].

Digital subtraction angiography not only demonstrates cerebral circulation and shared vessels but also has an important role prior to surgical management. In cases of shared arteries, coil embolization can be performed before the attempt at separation [19]. On the other hand, balloon occlusion of communications between venous structures can be performed to predict hemodynamic alterations after separation. In addition, vascular communications can be embolized to promote the formation of collaterals and facilitate surgical separation [20, 21].

## Cardiovascular and pulmonary systems

**Fig. 6:**
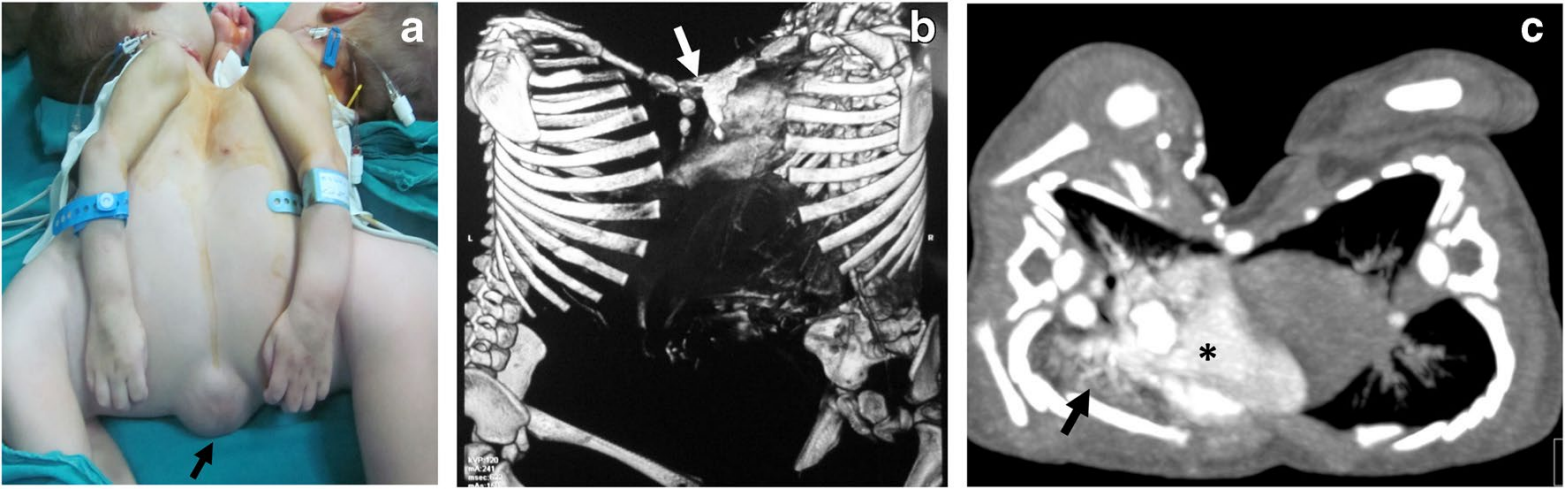
8-month-old thoracopagus conjoined twin boys. **a** Clinical photographic image shows thoracic and abdominal fusion with a small omphalocele (*arrow*). **b** 3-dimensional reconstruction of the bony skeleton shows sternal fusion (*arrow*).** c** Axial computed tomography of the thorax (mediastinal window) following contrast injection into the twin on the right shows opacification of the cardiac chambers of the injected baby only (*asterisk*), denoting separate cardiac chambers. Note the collapsed right lung with increased density (*arrow*)

Cardiac fusion is the major concern in conjoined twins, which can preclude their separation. The hearts are considered separate when heart rates are different or seen anatomically separate, as shown in the attached video (supplementary material 2). Echocardiography can accurately determine communications at the atrial or ventricular levels. Also, echocardiography identifies associated cardiac anomalies, which are common in all types of conjoined twins. CT is performed with contrast injected in one twin followed by the other (Fig. 6). The presence of intracardiac contrast in both hearts after injecting one twin confirms cardiac sharing. CT may be performed with electrocardiographic-gating for the accurate evaluation of cardiovascular anomalies [4]. Cardiac MRI and digital subtraction angiography are time-consuming and have been of limited additional value [5].

In addition to revealing cardiac anomalies and fused cardiac chambers, CT may demonstrate lung hypoplasia secondary to associated diaphragmatic hernia or tilted conjunction. Moreover, associated anomalies in the tracheobronchial tree (single fused trachea and esophagus or esophageal origin of the bronchi) can be readily detected. These anomalies are more common in parapagus and cephalopagus conjoined twins with a shared thoracic cavity [22].

## Hepatobiliary system

**Fig. 7:**
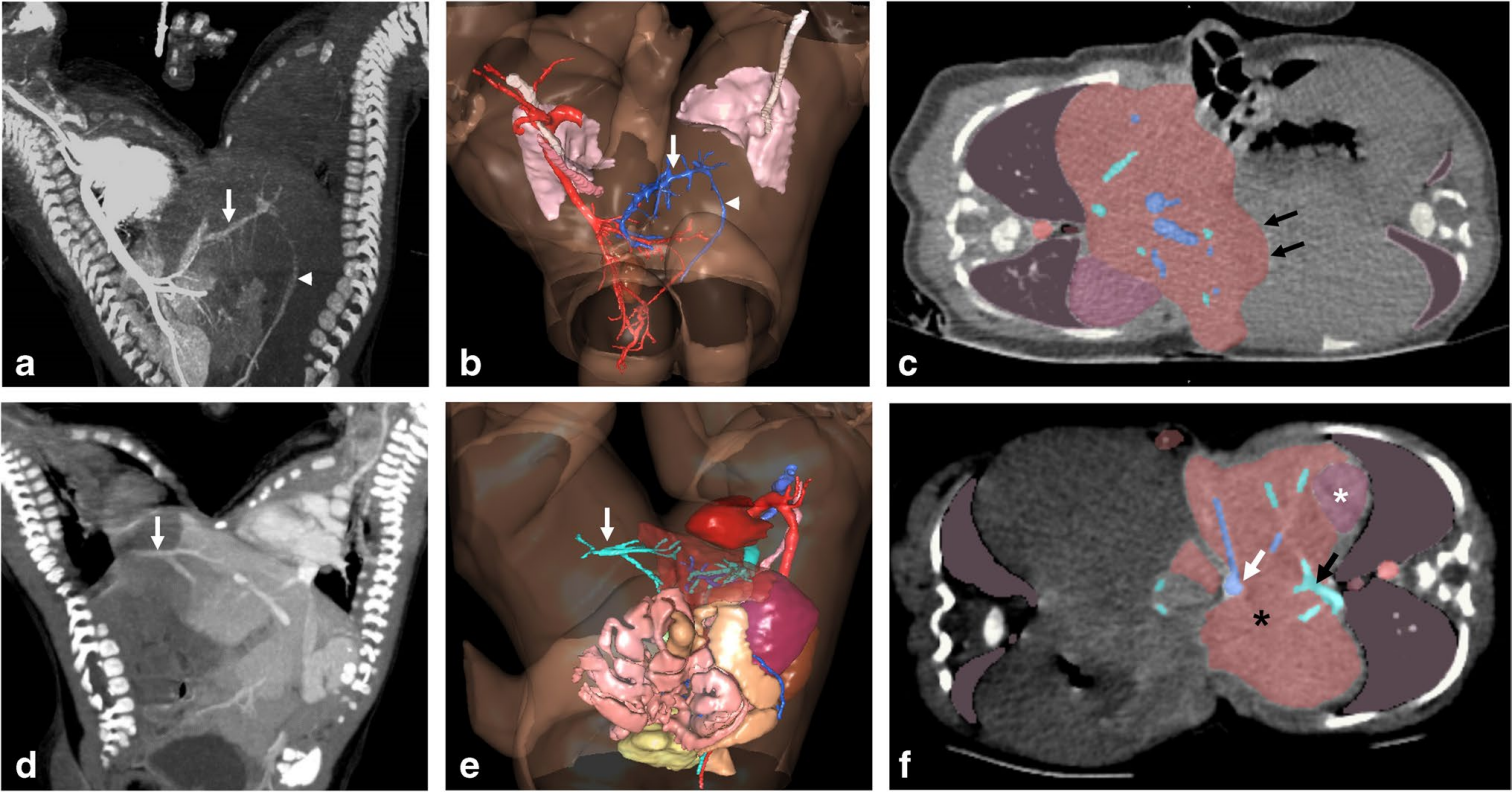
Computed tomography (CT) in 4-month-old ischiopagus conjoined twin boys following intravenous contrast injection of each twin on separate days. (**a**–**c**) are extracted following contrast injection of the right twin, while (**d–f**) are extracted following contrast injection of the left twin. **a, b** Maximum intensity projection (MIP) (**a**) and color-coded 3-dimensional (D) reconstruction) (**b**) show an abnormal venous channel (*arrow*) connecting the portal system of both twins with retrograde venous filling into the left twin *(arrowhead).* **c** Axial CT image (soft tissue window) with color overlay reveals the lobulated margin of the liver at the site of fusion (*arrows*). **d, e** Coronal CT (soft tissue window) (**d**) and 3-D with color overlay (**e**) show abnormal venous drainage into the right twin (a*rrow*) after the left twin was injected with contrast*.* **f** Axial CT image (soft tissue window) with color overlay shows the liver (*black* *asterisk*), spleen (*white asterisk*), hepatic vein (*black arrow*) and portal vein (*white arrow*) of the left twin. (Visible Patient®, Strasbourg, France. *Red* heart and aorta, *cyan* hepatic veins, *light orange* large intestine, *rosy brown *liver, *pink* lungs and trachea, *dark gray* lungs [lower lobes], *royal blue* portal veins, *light pink* small intestine, *mauve* spleen, *yellow* urinary bladder)

Hepatic fusion is another major concern in conjoined twins. Dynamic post-contrast CT should be acquired in multiple phases after contrast injection in each twin separately on two different days. A dynamic study demonstrates the arterial tree and shared vasculature with subsequent building of 3-D angiographic images. Post-contrast scans demonstrate the hepatic and bowel blood supply. The portovenous phase (hepatic phase) is optimum for the demonstration of the multiple irregular lobules of the liver at the site of fusion, which correspond to the surgical plane of separation (Fig. 7) [23]. It is crucial to show hepatic venous drainage into a discrete inferior vena cava. The absence of the inferior vena cava and abnormal venous drainage into the other twin are considered contraindications to separation [24, 25].

Preoperative evaluation of the biliary system can be challenging. The appearance of two distinct gall bladders and two duodenums indicates the existence of separate extrahepatic biliary systems. However, the presence of a single shared duodenum raises the suspicion of fusion of common bile ducts and even of the pancreatic ducts. Magnetic resonance cholangiography accurately demonstrates the biliary system of both twins and reliably excludes biliary sharing [23]. If there is still any doubt concerning biliary system sharing, a hepatobiliary iminodiacetic acid scintigraphy can be performed by injecting the tracer into each twin on two separate days. Separate hepatocyte uptake and excretion of the tracer imply separate biliary systems [26]. A shared biliary system is found in about a quarter of thoraco-omphalopagus twins [23].

## Gastrointestinal system

An upper gastrointestinal contrast study with follow through has been used in the evaluation of the gastrointestinal tracts of conjoined twins. Although it can reliably confirm separate gastric cavities in some cases, it has limited value in confirming bowel separation or precisely detecting the site of conjunction (supplementary material 3). Even when there is no fusion of bowel, conjoined twins with abdominal fusion (e.g., omphalopagus) usually share a common peritoneal cavity. Consequently, bowel loops from one twin may move freely through the shared cavity into the other twin, hindering precise determination of which bowel loops are shared [4]. Ventrally fused twins have variable degrees of gastrointestinal sharing. Thoraco-omphalopagus twins may have duodenal sharing with a single jejunum and ilium down to the level of Meckel’s diverticulum (at the site of the vitello-intestinal duct) [23]. In contrast, omphalo-ischiopagus twins commonly have bowel sharing distal to Meckel's diverticulum, with a single colon and rectum in 70%. The shared bowel loops usually have a dual blood supply from both twins [24].

Different types of anorectal anomalies can be encountered, especially in twins with pelvic conjunction (e.g., ischiopagus and pygopagus twins) [24–28], which may necessitate colostomy. In this instance, contrast studies through distal loopogram have been helpful in demonstrating suspected distal bowel sharing and rectal conjunction [27].

## Urogenital system

**Fig. 8:**
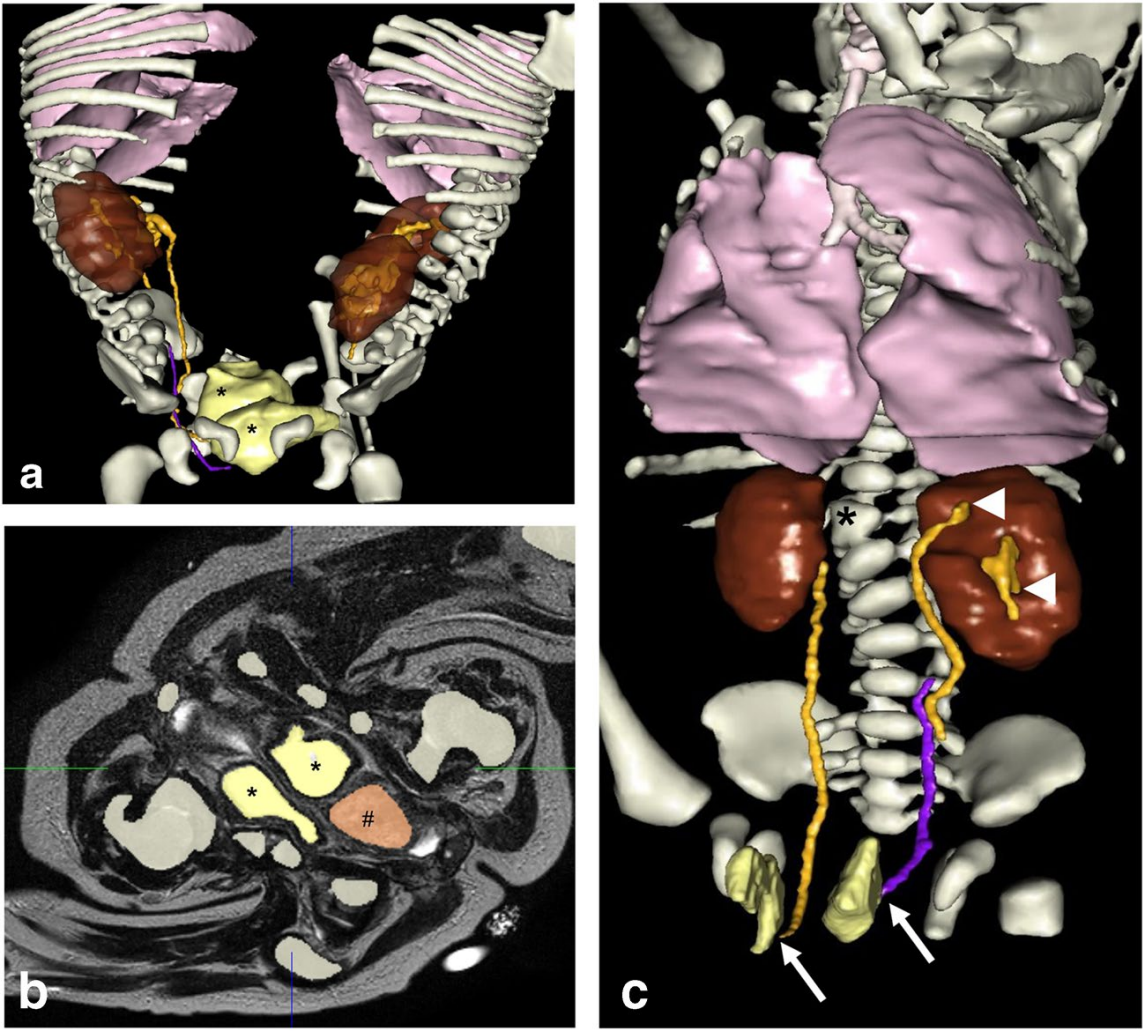
Imaging of 4-month-old ischiopagus conjoined twin boys (same patients as in Fig. 7). **a** Color-coded 3-dimensional (D) image extracted from a delayed phase computed tomography (CT) study. Each baby has two kidneys. There are two urinary bladders (*asterisks*) seen in the common pelvis. **b** Axial high resolution-T2-weighted magnetic resonance image with color overlay shows the anatomy of the conjoined pelvis. There are two urinary bladders (*asterisks*) and a single rectum (*#*). **c** Color-coded 3-D CT image of the left twin reveals each ureter draining into a separate bladder (*arrows*). The left kidney has a duplex collecting system (*arrowheads*) and proximal ureteric duplication. Note the presence of a hemivertebra (*asterisk*) (Visible Patient®, Strasbourg, France. *Ivory* bony skeleton, *brown* kidneys, *pink* lungs, *tan* rectum, *orange* ureters, *violet* ureter, *yellow* urinary bladders)

**Fig. 9:**
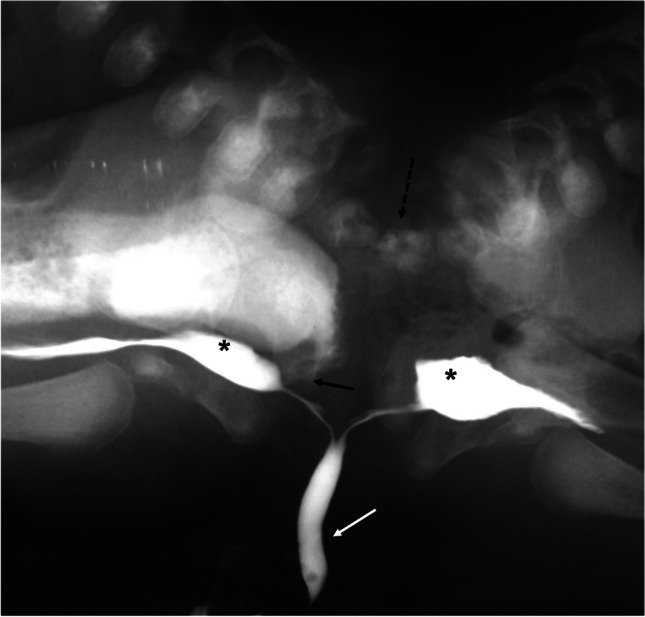
An anteroposterior pelvic radiograph obtained during micturating cystourethrography in 4-week-old pygopagus conjoined twin boys shows a single urethral channel (*white arrow*) with two separate urinary bladders (*asterisk*s). Note the vertebral fusion (*broken arrow*) and rectourethral fistula in the right twin (*black arrow*)

Multiple delayed urographic phases of CT should be acquired to demonstrate associated anomalies of the urinary tract, particularly those that involve the urinary bladder (number and orientation) and distal ureteric insertions. In twins with a shared pelvis (e.g., ischiopagus twins), each urinary bladder commonly drains a single ureter from each twin (Fig. 8) [5, 14, 24]. Micturating cystourethrography can reveal vesicoureteric reflux and associated anomalies of the urinary bladder and urethra and possible rectourethral fistula (Fig. 9) [4].

**Fig. 10:**
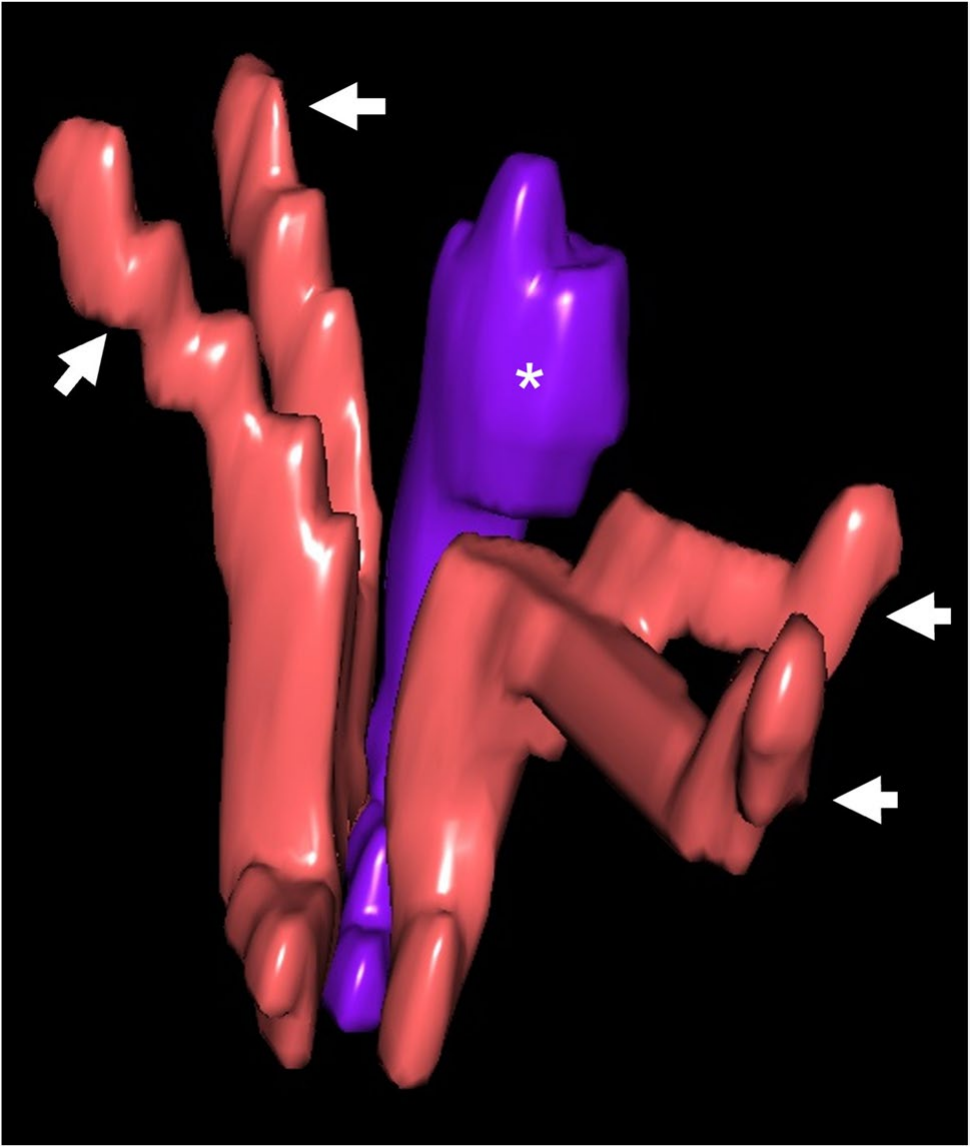
4-month-old ischiopagus conjoined twin boys with a common penis (same patients as in Figs. 7 and 8). Magnified 3-diemsnional view of the corporeal tissue extracted from axial high resolution T2-weighted magnetic resonance imaging. There are four corpora cavernosa (two belonging to each twin) *(arrows*) and a common corpus spongiosum (*asterisk*). (Visible Patient®, Strasbourg, France)

The advent of MR technology with the development of high-resolution thin section sequences that can be acquired in multiple planes has enabled accurate demonstration of pelvic anatomy, especially of the urethra and genital system (Figs. 8 and 10) [23].

## Musculoskeletal system

**Fig. 11:**
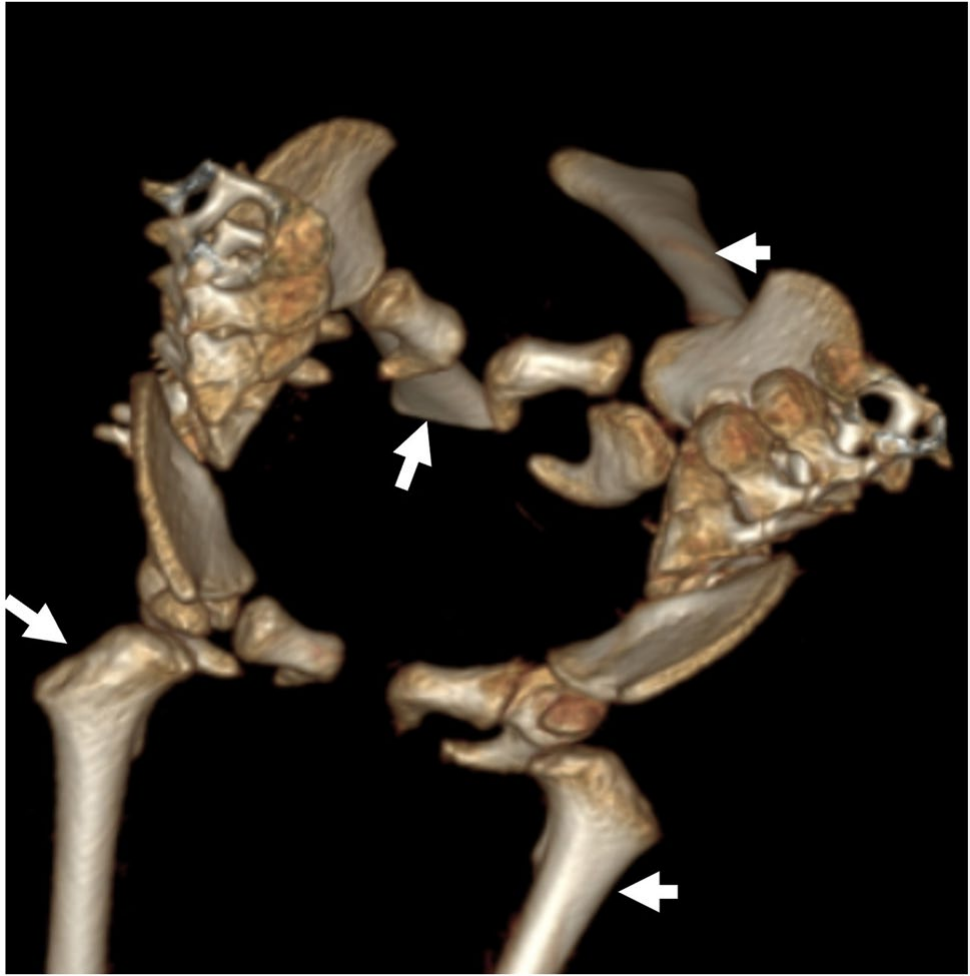
Pelvic fusion of 4-month-old ischiopagus tetrapus conjoined twin boys (same patients as in Figs. 7, 8 and 10). 3-dimensional computed tomography reconstruction of the bony pelvis shows that each baby has two normal lower limbs (*arrows*)

**Fig. 12:**
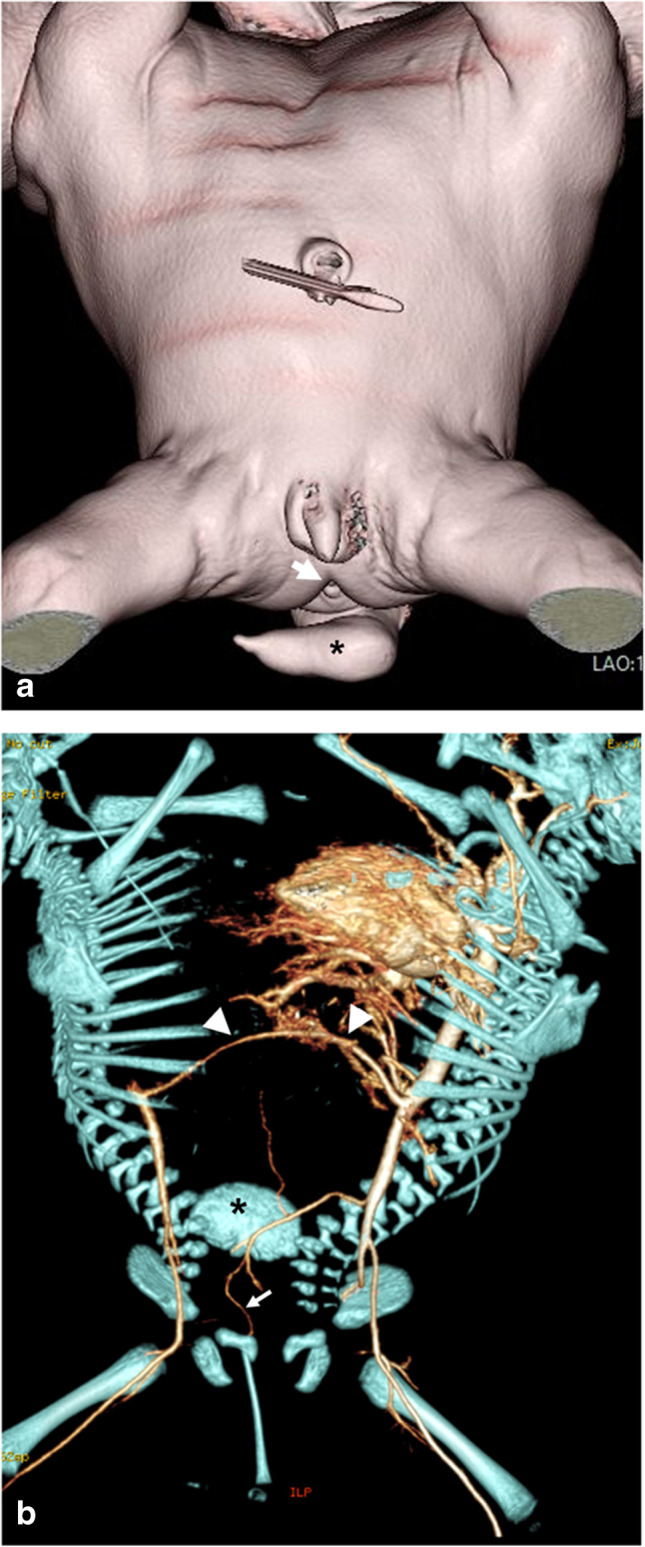
Computed tomography in 17-day-old ischiopagus tripus conjoined twin boys. **a** Surface-rendered image shows each baby to have one normal leg. There is a third deformed leg (*asterisk*) attached posteriorly to the conjoined pelvis. Note the single anal orifice (*arrow*) and single penis and scrotum. **b** 3-dimensional reconstruction of the skeleton and arterial tree following contrast injection of the left twin reveals a common iliac bone posteriorly (*asterisk*) and short bones of the deformed limb supplied by a small branch (*arrow*) arising from a hypoplastic right common iliac artery*.* A shared superior mesenteric artery (*arrowheads*) connects the aortas of both twins

CT with 3-D reconstruction is helpful for the preoperative visualization of the bony skeleton and the planning of osteotomies. Twins with pelvic sharing usually have four lower extremities attached to their fused pelvis (tetrapus) (Fig. 11). However, they may have only two (bipus) or three (tripus) lower limbs. In tripus twins, each baby has a normal leg and a deformed leg attached posteriorly to the conjoined pelvis (Fig. 12) [23]. CT arteriography can demonstrate the arterial supply of all limbs, including the shared limb, which is crucial for surgical reconstruction (Fig. 12) [4, 5].

## Recent advances and structured reporting

Recently, 3-D virtual and printed models have been found to play a crucial role in preoperative surgical mapping. Different structures can be visualized in different colors and printed with different materials (Figs. 7, 8, and 10). They help the surgeons understand the radiological anatomy of the bones and soft tissues thus increasing the effectiveness of preoperative multidisciplinary meetings. Moreover, 3-D-printed models offer the surgeons a physical structure that can be held, which increases their tactile perception. The 3-D-printed models enable simulation rehearsal for surgeons and anesthesiologists, allowing them to practice how to position the twins and where the surgical plane should be. This may increase their confidence during the operation and minimize the time of surgery and related risks. Additionally, 3-D-printed models help better parent counseling, enabling surgeons to communicate effectively with parents and discuss the surgical strategy, which eventually promotes parents’ engagement and satisfaction [29, 30].

Preoperative planning for separation of the different types of conjoined twins has been aided by 3-D virtual and printed models. For better identification of the complex cranial anatomy, 3-D printed transparent acrylic and ceramic models have been developed especially for the intracranial vessels. These models are valuable for planning craniotomy and designing bone grafts when aiming to rebuild the expected cranial defects [18]. Additionally, 3-D modeling allows better visualization of the thoracic cage and sternum. This is crucial in twins who have significant sternal fusion, since exocardia is more common following separation [21]. Estimation of hepatic volume and optimum plane of hepatic separation can also be identified [29].

Virtual and augmented reality are rapidly developing and being implemented in various medical fields. Based on CT and MRI-extracted 3-D models, virtual reality (VR) allows surgeons to be immersed in a digital environment simulating the operating theater using a VR headset [31]. Moreover, VR enables surgeons from different institutions to collaborate and practice surgery in a VR room. VR has been used in the planning of separation of craniopagus and thoracopagus twins with successful results [32–34].

Structured reports provide more clinically relevant information to support decision-making [14]. Due to the complexity of conjoined twins, the report should be structured as follows: (1) Start with a clear statement about orientation of the twins and description of the radiologic labeling if used (e.g., olive capsule). (2) Soft tissue and visceral fusion and associated congenital anomalies should be properly described as mentioned in the previous sections: (a) central nervous system, (b) cardiovascular system, highlighting the presence or absence of shared perfusion and vascular connections, (c) hepatobiliary system, (d) gastrointestinal system and (e) genitourinary system. (3) In the skeletal system, the site and extent of skeletal fusion and the associated skeletal anomalies should be clearly described. (4) Finally, images of complex radiological findings should be attached to the report since they are very helpful to surgeons.

## Conclusion

Advancements in medical imaging and postprocessing techniques play a pivotal role in the management of conjoined twins. After delivery, US and echocardiography are initially performed for all conjoined twins. Further imaging modalities should be selected according to the site of conjunction. A properly tailored imaging strategy accurately defines the shared organs and associated anomalies, which are considered the backbone for successful separation.

### Supplementary Information

Below is the link to the electronic supplementary material.

Supplementary file1 (JPG 873 kb)

Supplementary file2 (MP4 2988 kb)

Supplementary file3 (JPG 1173 kb)

## Data Availability

The datasets used during the current study are available from the corresponding author on reasonable request.

## References

[1] Barth RA, Filly RA, Goldberg JD (1990). Conjoined twins: prenatal diagnosis and assessment of associated malformations. Radiology.

[2] Spencer R (2000). Theoretical and analytical embryology of conjoined twins: part I: embryogenesis. Clin Anat (New York, N.Y.).

[3] Kaufman MH (2004). The embryology of conjoined twins. Childs Nerv Syst.

[4] McHugh K, Kiely EM, Spitz L (2006). Imaging of conjoined twins. Pediatr radiol.

[5] Kingston CA, McHugh K, Kumaradevan J (2001). Imaging in the preoperative assessment of conjoined twins. Radiographics.

[6] MacKenzie TC, Crombleholme TM, Johnson MP (2002). The natural history of prenatally diagnosed conjoined twins. J Pediatr Surg.

[7] Watson SG, McHugh K (2015). Conjoined twins: radiological experience. Semin Pediatr Surg.

[8] Weaver MP (1953) Tlatilco and the Preclassic Cultures of the New World Viking Fund Publ Anthro (19) Wenner-Gren Foundation for anthropological Research. https://books.google.com.eg/books/about/Tlatilco_and_the_Pre_classic_Cultures_of.html?id=1ahoAAAAMAAJ&redir_esc=y

[9] Kennedy GE (2001). The 3,000-year history of conjoined twins. West J Med.

[10] Patankar T, Fatterpekar GM, Prasad S (2000). Fetus in fetu: CT appearance–report of two cases. Radiology.

[11] Sharma G, Mobin SS, Lypka M, Urata M (2010). Heteropagus (parasitic) twins: a review. J Pediatr Surg.

[12] Spencer R (1996). Anatomic description of conjoined twins: a plea for standardized terminology. J Pediatr Surg.

[13] Spitz L, Stringer MD, Kiely EM (1994). Separation of brachio-thoraco-omphalo-ischiopagus bipus conjoined twins. J Pediatr Surg.

[14] Eley KA, Rossi-Espagnet MC, Schievano S (2021). Multiparametric imaging for presurgical planning of craniopagus twins: the experience of two tertiary pediatric hospitals with six sets of twins. Radiology.

[15] Mathew RP, Francis S, Basti RS (2017). Conjoined twins: role of imaging and recent advances. J Ultrason..

[16] Spetzler RF, Martin NA (1986). A proposed grading system for arteriovenous malformations. J Neurosurg.

[17] Goldman-Yassen AE, Goodrich JT, Miller TS, Farinhas JM (2020). Preoperative evaluation of craniopagus twins: anatomy, imaging techniques, and surgical management. AJNR Am J Neuroradiol.

[18] Yokota C, Kagawa N, Bamba Y, et al (2021) Successful neurosurgical separation of conjoined spinal cords in pygopagus twins: illustrative cases. J Neurosurg: Case Lessons 1: 10.3171/case218 10.3171/CASE218 PMC9241251 35854707

[19] Rutka JT, Souweidane M, ter Brugge K (2004). Separation of craniopagus twins in the era of modern neuroimaging, interventional neuroradiology, and frameless stereotaxy. Childs Nerv Syst.

[20] Alokaili RN, Ahmed ME, Al Feryan A (2015). Neurointerventional participation in craniopagus separation. Interv Neuroradiol.

[21] Heuer GG, Madsen PJ, Flanders TM (2019). Separation of craniopagus twins by a multidisciplinary team. N Engl J Med.

[22] Spencer R (2003). Conjoined twins: developmental malformations and clinical implications.

[23] AbouZeid AA, Mohammad SA, Radwan AB (2022). Ventrally fused conjoined twins (omphaloischiopagus): a roadmap to successful separation. Eur J Pediatr Surg Rep.

[24] Cywes S, Millar AJ, Rode H, Brown RA (1997). Conjoined twins–the Cape Town experience. Pediatr Surg Int.

[25] O'Neill JA, Holcomb GW, Schnaufer L (1998). Surgical experience with thirteen conjoined twins. Ann Surg.

[26] Meyers RL, Matlak ME (2002). Biliary tract anomalies in thoraco-omphalopagus conjoined twins. J Pediatr Surg.

[27] AbouZeid, AAZ, Mohammad SA (2022) Conjoined twins. In: Atlas of Anorectal Anomalies. Springer, Cham. 10.1007/978-3-031-10282-0_19

[28] AbouZeid AA, Mohammad SA, Zaki AM, AbdelHay S (2022). Separation of pygopagus conjoined twins: what has changed after 15 years. J Pediatr Surg.

[29] Wood BC, Sher SR, Mitchell BJ (2017). Conjoined twin separation: integration of three-dimensional modeling for optimization of surgical planning. J Craniofac Surg.

[30] Inserra A, Borro L, Spada M (2020). Advanced 3D “modeling” and “printing” for the surgical planning of a successful case of thoraco-omphalopagus conjoined twins separation. Front Physiol.

[31] Rufai SR, Gore S, Handley SE (2021). Enhanced neuro-ophthalmologic evaluation to support separation of craniopagus twins. J Surg Case Rep.

[32] Khor WS, Baker B, Amin K (2016). Augmented and virtual reality in surgery-the digital surgical environment: applications, limitations and legal pitfalls. Ann Transl Med.

[33] Juhnke B, Mattson AR, Saltzman D (2019). Use of virtual reality for pre-surgical planning in separation of conjoined twins: a case report. Proc Inst Mech Eng H.

[34] Ribeiro G, Werner H, Lopes J (2021). Craniopagus twin: pre- and post-natal 3-dimensional virtual and physical models and virtual navigation created with free or open source software-an option for low-resource centers. Childs Nerv Syst.

