# Supplementary figures and images for: Postnatal imaging of conjoined twins: a customized multimodality approach
Source: Pediatr Radiol. 2023 Jul 19;53(11):2291–304. doi: 10.1007/s00247-023-05709-3 (PMC10562291; doi:10.1007/s00247-023-05709-3)

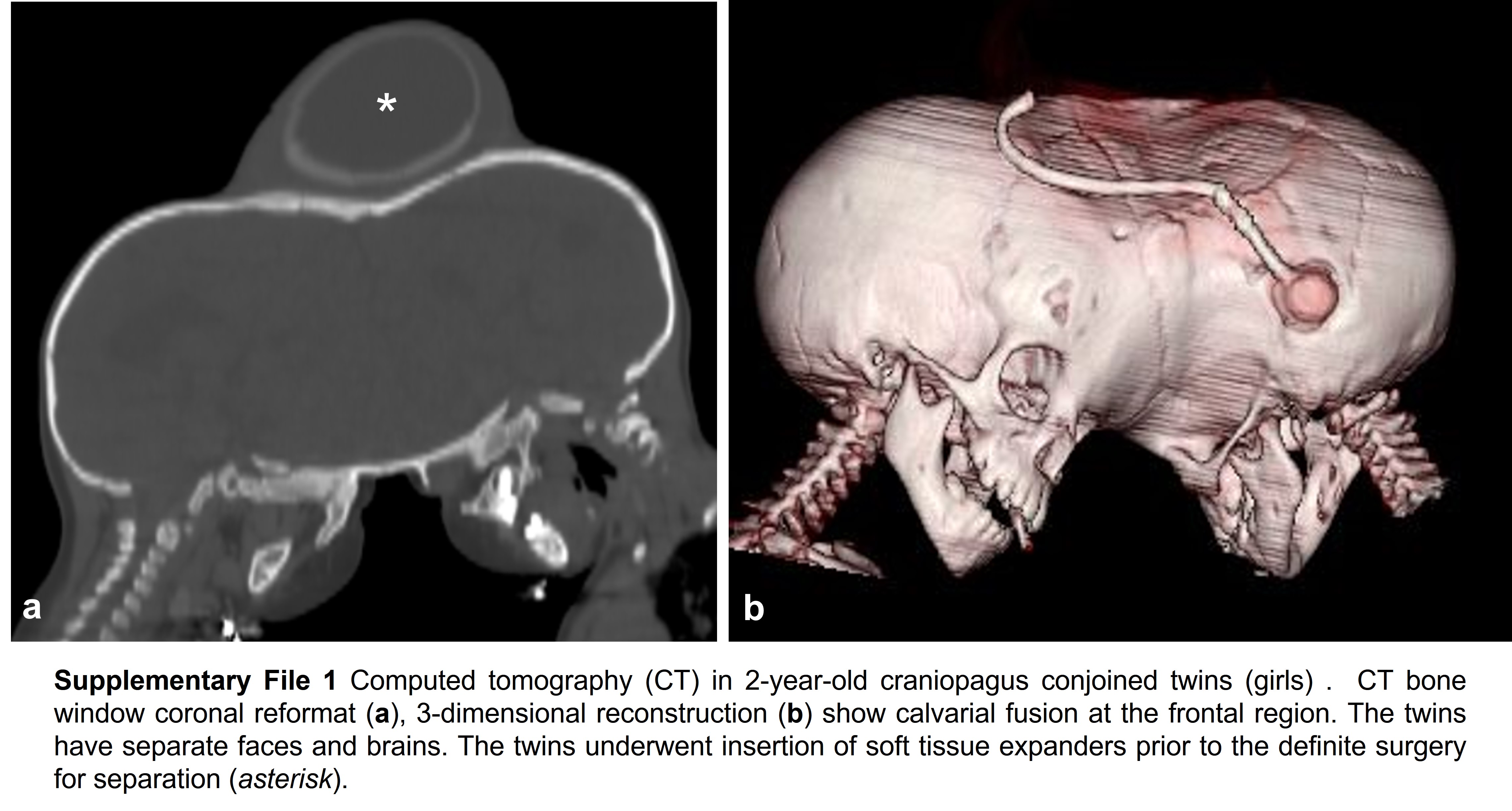

Supplement: Supplementary file 1 — Supplementary file1 (JPG 873 kb) [file 247_2023_5709_MOESM1_ESM.jpg]

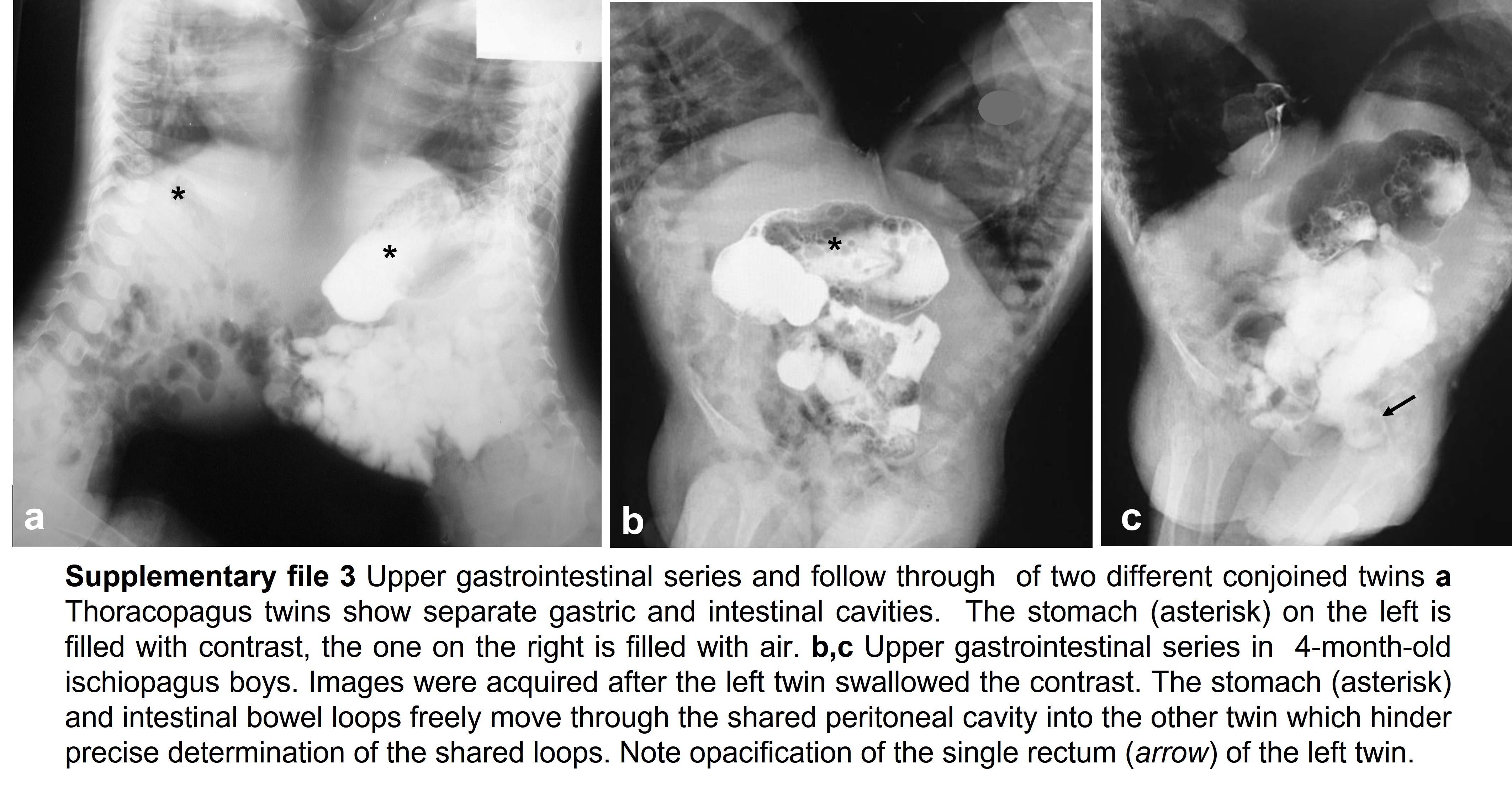

Supplement: Supplementary file 3 — Supplementary file3 (JPG 1173 kb) [file 247_2023_5709_MOESM3_ESM.jpg]
